# Risk factors and prognosis of symptomatic postpartum ovarian vein thrombosis in 2 French prospective cohort studies

**DOI:** 10.1016/j.rpth.2025.102849

**Published:** 2025-04-09

**Authors:** Eloïse Laouenan, Claire de Moreuil, Sara Robin, Karine Morcel, François Anouilh, Cécile Tromeur, Francis Couturaud, Emmanuelle Le Moigne

**Affiliations:** 1 Brest University (Univ Brest), Institut National de la Santé et de la Recherche Médicale (INSERM), Unit Research U1304-GETBO (Groupe d'Etude de la Thrombose de Bretagne Occidentale), Brest, France; 2 Département de Gynécologie–Obstétrique, Centre Hospitalier Universitaire (CHU) de Brest, Brest, France; 3 French Clinical Research Infrastructure Network (F-CRIN), INvestigation Network On Venous Thrombo-Embolism (INNOVTE), Saint-Etienne, France; 4 Service de Médecine Interne, Centre Hospitalier Universitaire (CHU) de Brest, Brest, France; 5 Faculté de Maïeutique, Université de Bretagne Occidentale (UBO), Brest, France; 6 Service de Pneumologie, Centre Hospitalier Universitaire (CHU) de Brest, Brest, France

Ovarian vein thrombosis (OVT), an unusual site of venous thromboembolism (VTE), occurs mostly during gynecological surgery, cancer, and postpartum. Symptomatic postpartum ovarian vein thrombosis (sPOVT) is distinguished from other medical contexts by a lower prevalence (OVT occurred in 0.06% of deliveries vs 37.0% for pelvic cancer), younger age, obstetric-related risk factors (endometritis and cesarean delivery), abdominal pain and fever in 100.0% for obstetrics vs 17.0% for pelvic cancer, more serious complications (extension to the inferior vena cava or left renal vein and concomitant pulmonary embolism [PE] or deep vein thrombosis [DVT]), and potentially fewer recurrences (3.6% after OVT in the postpartum period vs 17.1% after OVT for pelvic cancer) [[1], [2], [3]]. However, data on the long-term risk of recurrent VTE after sPOVT remain limited, and recommendations for the duration of anticoagulant treatment vary from 6 weeks to 6 months [[4], [5], [6]]. The present study aimed to evaluate (i) the risk factors for sPOVT; (ii) the short-term prognosis, including thrombus extension to the inferior vena cava or left renal vein and association with PE or DVT; (iii) the risk of recurrent VTE after anticoagulation discontinuation; and (iv) the therapeutic modalities.

This descriptive study included all documented cases of sPOVT identified from 2 French multicenter prospective cohorts: the HEMOrrhage and venous THromboEmbolism in PostPartum—HEMOTHEPP study (NCT 02443610), which enrolled 20,238 pregnant women from 2015 to 2019 [7], and the Étude des Déterminants et Interactions de la THrombose veineuse—EDITH study (NCT 04297085), which consecutively enrolled 7102 adult patients with VTE since 2010 [8]. Diagnoses of sPOVT, VTE recurrence, and maternal death were reviewed and validated by an independent clinical events committee (S.R., C.D.M., and F.C.) blinded to the study population characteristics. Ethical approval was obtained, and written informed consent was obtained from all participants in both studies.

Thirteen women with sPOVT were identified (8 from the HEMOTHEPP study and 5 from the EDITH study). In all the women, the diagnosis of sPOVT was based on the association of clinical and biological symptoms (abdominal pain, elevated C-reactive protein, and/or fever) and the presence of a filling defect within the ovarian veins on CT scan. The median time to diagnosis was 8 days postpartum (IQR, 4.5-12.0) (Table). Clinical characteristics included age ≥35 years (*n* = 6; 46.2%), a family history of VTE (*n* = 4; 30.4%), obesity (*n* = 3; 23.1%), and comorbidities (*n* = 1; 7.7%, valvular cardiopathy) (Table). All women were tested for thrombophilia: none had factor (F)V Leiden, G20210A prothrombin gene variant, and antiphospholipid syndrome. No woman had a personal history of VTE. Obstetric-related risk factors included symptomatic endometritis confirmed by the presence of bacterial colonization in a vaginal swab (*n* = 9; 69.2%), preterm delivery (*n* = 4; 30.4%), multiple pregnancy (*n* = 3; 23.1%), and cesarean delivery (*n* = 3; 23.1%) (Table). All women presented with abdominal pain and elevated C-reactive protein, and 9 of them had fever.

**Table:** Baseline characteristics of the 13 women, thromboprophylaxis, post-diagnosis management and follow-up of symptomatic postpartum ovarian vein thrombosis.

Patient	A	B	C	D	E	F	G	H	I	J	K	L	M	Total *N* (%)
Preexisting risk factors														
Age ≥ 35 y			x			x	x	x		x		x		6 (46.1)
Body mass index ≥ 30 kg/m^2^	x								x	x				3 (23.1)
Smoking			x			x								2 (15.4)
Antenatal parity ≥3	x									x				2 (15.4)
Cardiac, renal, or inflammatory bowel disease^a^												x^b^		1 (7.7)
Family history of VTE			x						c	x	x		x	4 (30.8)
Previous VTE														0 (0)
Thrombophilia (Factor (F)V Leiden and prothrombin gene variant)														0 (0)
Pregnancy-related risk factors														
Multiple pregnancy				x			x		x					3 (23.1)
Placental vascular disease				x										1 (7.7)
Gestational diabetes									x		x			2 (15.4)
Delivery-related risk factors														
Preterm delivery (<37 wk gestation)				x	x		x	x						4 (30.8)
Stillbirth								x						1 (7.7)
Cesarean delivery					x		x		x					3 (23.1)
Postpartum hemorrhage (≥ 500 mL)				x	x								x	3 (23.1)
Postpartum infection				x^d^	x^d^	x^d^	x^e^	x^d^	x^e^	x^d^	x^d^		x^f^	9 (69.2)
Postpartum surgery							x				x			2 (15.4)
Postpartum thromboprophylaxis and time to occurrence of sPOVT														
Postpartum thromboprophylaxis (day to day after delivery)	0-8							0-2	0-5		3-13			4 (30.8)
Time to occurrence of sPOVT (d after delivery)	8	9	24	1	5	7	10	2	11	13	20	4	7	8.0 (4.5-12.0)
Anticoagulant treatment														
Only LMWH (L) or fondaparinux (F)	L										F	L	L	4 (30.8)
LMWH (L) or fondaparinux (F) then VKA relay		L		L			L			L				4 (30.8)
LMWH (L) or fondaparinux (F) then DOAC relay			F		F	L		L	L					5 (38.5)
Total anticoagulation duration (mo)	3	6	6	9	3	3	6	3	6	6	1.5	3	3	3.0 (3.0-6.0)
Concomitant antibiotics		x		x	x	x	x	x		x	x	x	x	10/12 (83.3)
Imaging follow-up		x	x	x			x	x	x	x		x		8/11 (72.7)
Extension to the inferior vena cava or left renal vein at diagnosis	x		x	x						x				4 (30.8)
sPOVT + PE			x	x										2 (15.4)
sPOVT + other VTE			x											1 (7.7)
Recurrent VTE											x			1 (7.7)
Death												x		1 (7.7)

DOAC, direct oral anticoagulant; LMWH, low-molecular-weight heparin; PE, pulmonary embolism; sPOVT, symptomatic postpartum ovarian vein thrombosis; VKA, vitamin K antagonists; VTE, venous thromboembolism.

a Ischemic heart disease, congenital heart disease, cardiac failure, cardiac arrythmias, cardiomyopathy, glomerular disease, renal tubulointerstitial disease, renal failure, ulcerative colitis, Crohn’s disease, or nonspecific inflammatory bowel disease.

b Valvular cardiopathy.

c First-degree sudden death at 42.

d Endometritis.

e Cesarean scar infection.

f Urinary tract infection.

Retrospective application of the Royal College of Obstetricians and Gynaecologists (RCOG) risk prediction score for postpartum VTE [9] classified 1 patient as at low risk of VTE, while the others were at intermediate risk with at least 3 risk factors, indicating postpartum thromboprophylaxis for at least 10 days. Of the 4 (30.8%) women who received postpartum thromboprophylaxis, 2 had sPOVT in the context of obesity without dose adjustment in 1 woman and at the start of thromboprophylaxis in the other, and 2 others had sPOVT after thromboprophylaxis was discontinued.

At sPOVT diagnosis, 4 women (30.8%) presented thrombus extension to the left renal vein or inferior vena cava; 1 of these had concomitant PE and another had contralateral renal vein thrombosis. None had concomitant lower limb DVT.

Regarding therapeutic anticoagulation, the median duration of treatment was 3 months (IQR, 3.0-6.0) with no reports of clinically relevant nonmajor bleeding or major bleeding. Four women (30.8%) received low-molecular-weight heparin (LMWH) or fondaparinux alone and 9 (69.2%) received LMWH with early switching to vitamin K antagonists in 4 and to direct oral anticoagulants in 5. Ten women (83.3%) were treated with concomitant antibiotics.

During a median follow-up of 6.1 years (IQR, 3.0-7.3) after therapeutic anticoagulation discontinuation, 1 woman had a recurrent VTE at 5 years of follow-up (muscular vein thrombosis after surgery) and another woman died of Hodgkin’s lymphoma without a recurrent VTE 5 years after sPOVT.

Our findings warrant several comments. First, consistent with 2 previous reviews, proximal thrombus extension and/or concomitant PE or DVT are frequent complications of sPOVT [1,10]. This observation highlights the need for early diagnosis and for therapeutic anticoagulation administered for at least 3 months, as recommended for proximal DVT and PE [11]. Second, to our knowledge, this is the first study to assess the risk of recurrent VTE after sPOVT during long-term follow-up. The apparent low risk of recurrent VTE after sPOVT (ie, only 1 provoked recurrence at 5 years) is consistent with the low risk of recurrence observed in women with hormone-related VTE [1,2,12] and supports treating sPOVT similarly to provoked PE or DVT [11] (ie, for a maximum period of 6 months), as recommended by the United Kindom guidelines [4]. Third, the frequent association between sPOVT and endometritis observed in our study and elsewhere [1,3,13,14] suggests that more systematic VTE evaluation is needed in cases of persistent fever or abdominal pain despite adequate antibiotic treatment. In addition, reassessment of VTE risk is advisable if endometritis occurs. Fourth, only one third of women classified at intermediate risk of VTE according to the RCOG score received thromboprophylaxis, which highlights the underestimation of VTE risk and undertreatment also reported by Chau et al. [15]. The use of a postpartum VTE risk score, recommended since 2008 [16], is valuable in clinical practice for thrombosis risk assessment and subsequent thromboprophylaxis [5]. The implementation of computerized clinical decision support systems may improve compliance with these recommendations [17].

In conclusion, sPOVT is a rare but potentially serious postpartum complication that requires early diagnosis and adequate therapeutic anticoagulation for a minimum of 3 months and a maximum of 6 months. Given the low prevalence of the disease, the study’s findings are based on a small sample size, which may restrict the generalizability of the results. Further studies on optimal therapeutic and prophylactic strategies are needed.
